# Pharmacy Benefit Manager Market Concentration for Prescriptions Filled at Retail Pharmacies by State and Payer Type

**DOI:** 10.1001/jamahealthforum.2025.6546

**Published:** 2026-02-06

**Authors:** Dima Mazen Qato, Yugen Chen, Karen Van Nuys

**Affiliations:** 1 Program on Medicines and Public Health, Alfred E. Mann School of Pharmacy and Pharmaceutical Sciences, University of Southern California, Los Angeles; 2 Leonard D. Schaeffer Center for Health Policy and Economics, University of Southern California, Los Angeles; 3 Department of Economics, College of Letters, Arts and Sciences, University of Southern California, Los Angeles; 4 Sol Price School of Public Policy, University of Southern California, Los Angeles

## Abstract

This cross-sectional study examines whether and how pharmacy benefit manager concentration varies across states and payer types.

## Introduction

Pharmacy benefit managers (PBMs), which serve as intermediaries between health insurers and pharmacies, are under investigation by the Federal Trade Commission for potential antitrust violations and anticompetitive practices contributing to high out-of-pocket costs and pharmacy closures.^1^ In our prior study we found that nationally, PBM market concentration for retail prescriptions varied by payer type, was most concentrated in Medicare Part D, and the top 3 PBMs (Caremark, Express Scripts, and Optum Rx) accounted for more than 75% of the market for all payer types.^2^ Despite the critical role of states in PBM regulations,^3^ and evidence PBM markets vary across geographies,^4^ information on whether and how PBM concentration varies across states is limited.

## Methods

We used IQVIA’s National Prescription Audit PayerTrak, which includes 92% of prescription fills at US retail pharmacies, including information on the processing PBM, payer type (commercial insurance, Medicare Part D, and Medicaid managed care), and state where the prescription was dispensed (eMethods in Supplement 1). We calculated PBM market concentration for all retail prescriptions filled in 2023 (primary outcome) and total market share for the top 3 PBMs overall and by payer type for each state (secondary outcomes). PBM market concentration was defined using the Herfindahl-Hirschman Index (HHI). Highly concentrated markets were defined as those with an HHI above 2500, consistent with US Department of Justice merger guidelines in 2023.^5^

This cross-sectional study followed the STROBE reporting guidelines. The University of Southern California institutional review board did not consider this study human participant research.

## Results

This study included 90% of prescriptions (3.6 billion) filled at US retail pharmacies and adjudicated by 91 PBMs in 2023. Overall, HHI varied substantially across states, ranging from 1443 in Idaho to 4273 in Hawaii, with 12 states identified as having highly concentrated PBM markets (Figure). While only 10 states were found to be highly concentrated for all payer types, 46 states were considered highly concentrated for at least Medicaid managed care or Part D. Specifically, 21 states were considered highly concentrated in both Part D and Medicaid; 17 states were highly concentrated for only Medicare (eg, California) or Medicaid (eg, Florida) PBM markets.

**Figure.**
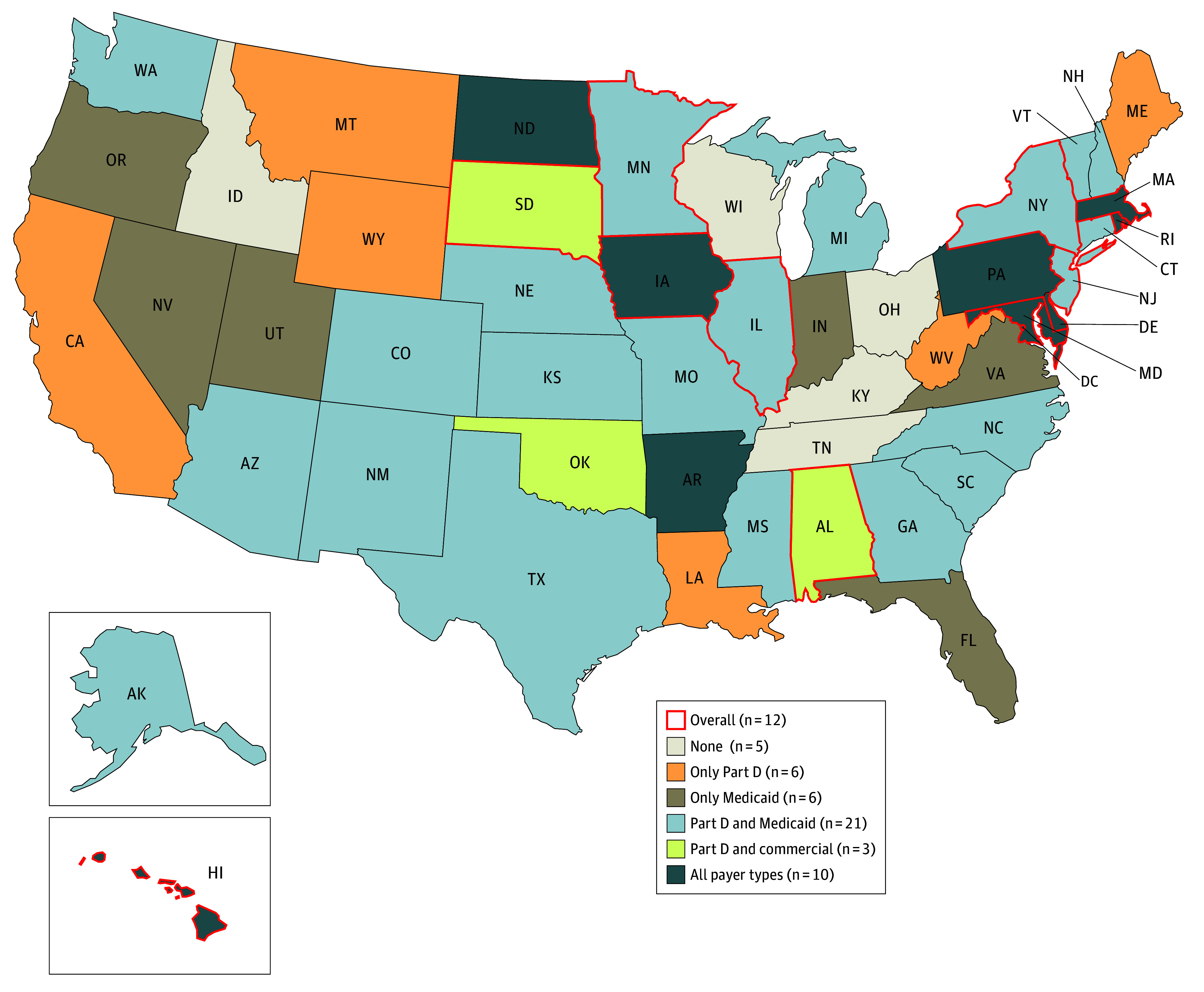
Pharmacy Benefit Manager (PBM) Market Concentration by State and Payer Type Concentration was measured by Herfindahl-Hirschman Index (HHI; sum of each PBM’s share of retail prescriptions squared). Highly concentrated was defined as HHI above 2500. Retail prescriptions (10% out of 3.6 billion claims) filled through other methods of payments for which PBMs are not involved, including Medicaid fee-for-service, the Medicaid Children’s Health Insurance Program, and cash payment without insurance, were excluded. Certain PBMs (eg, Aetna with Caremark) were combined based on outsourcing and ownership relationships (eMethods in Supplement 1). Iowa, Delaware, Maryland, Hawaii, Rhode Island, Massachusetts, New Jersey, Illinois, New York, Minnesota, Alabama, and South Dakota had HHI above 2500 overall. HHI ranged from 1443 (Idaho) to 4273 (Hawaii), Part D HHI ranged from 2041 (Wisconsin) to 4105 (Hawaii), Medicaid HHI ranged from 1644 (Alabama) to 9205 (Kentucky), and commercial insurance HHI ranged from 1327 (Idaho) to 3856 (Hawaii). State Medicaid authorities in Ohio and Kentucky contracted with a single pass-through PBM for all Medicaid managed care business throughout the state.

States with highly concentrated PBM markets accounted for 16.1%, 93.7%, and 74.8% of all retail prescriptions paid for through commercial insurance (13 states), Medicaid managed care (37 states), and Medicare Part D (40 states), respectively (Table). In nearly all of these highly concentrated states, the top 3 PBMs accounted for the majority of retail prescriptions for all payer types. In fact, one of these top 3 PBMs accounted for at least 50% of the market in many cases (ie, Caremark alone accounted for 83.1% and 71.8% of the Medicaid managed care market in Hawaii and Illinois, respectively).

**Table.** Market Share for the Top 3 Pharmacy Benefit Managers (PBMs; Caremark, Optum Rx, and Express Scripts) for Prescription Fills at Retail Pharmacies by State and Payer Type in the US in 2023^a^^,^^b^

State	Commercial insurance (1 824 166 912 total fills)			Medicaid managed care (336 980 256 total fills)^c^			Medicare Part D (1 117 485 056 total fills)		
	HHI	Total fills, %	Fills at top 3 PBMs,%	HHI	Total fills, %	Fills at top 3 PBMs, %	HHI	Total fills, %	Fills at top 3 PBMs, %
Alabama	3141	2.1	82.4	1644	0.1	56.0	2681	2.2	83.0
Alaska	1957	0.2	72.3	3035	0.0	74.2	2869	0.1	78.3
Arizona	1810	1.8	71.0	4876	3.5	98.0	2768	1.8	79.3
Arkansas	2525	1.4	69.7	4722	0.3	92.9	2671	1.4	78.0
California	2144	6.8	69.9	1992	0.6	61.0	2609	8.2	81.0
Colorado	1566	1.3	63.7	2749	0.3	69.8	3058	1.0	76.4
Connecticut	2073	1.1	73.4	2512	0.2	77.7	3139	1.2	86.1
Delaware	2725	0.3	83.1	4857	0.7	65.2	3039	0.3	85.2
Florida	2274	6.9	77.6	3615	6.2	67.0	2320	7.8	66.3
Georgia	1923	3.9	67.3	3315	2.9	25.2	2675	3.2	68.5
Hawaii	3856	0.3	85.7	7097	0.6	97.5	4105	0.3	88.8
Idaho	1327	0.5	46.9	2318	0.1	53.3	2133	0.5	62.5
Illinois	2416	3.8	79.3	5763	6.2	97.0	2673	3.8	81.5
Indiana	1492	2.2	60.6	3199	4.6	29.9	2264	2.2	62.9
Iowa	2668	1.0	77.1	4610	1.9	52.6	2727	1.2	81.4
Kansas	1916	1.2	73.3	4807	0.9	96.2	2737	1.0	73.7
Kentucky	1668	1.6	59.1	NA	NA	NA	2265	2.1	49.8
Louisiana	2413	1.7	77.6	2309	5.3	71.3	2667	1.9	64.8
Maine	1569	0.4	58.9	2500	0.1	74.1	3187	0.5	76.3
Maryland	3471	1.9	84.9	2967	2.6	41.3	3164	1.3	85.9
Massachusetts	2704	2.1	83.6	3529	2.7	97.4	3608	2.2	88.2
Michigan	2309	3.2	78.9	2579	5.9	68.7	2822	3.5	83.9
Minnesota	2353	1.4	72.5	4573	2.2	69.2	3087	1.5	81.8
Mississippi	1839	1.2	63.6	3375	1.1	96.6	2859	1.3	64.5
Missouri	1724	2.1	68.9	3672	0.3	79.1	2535	2.2	72.5
Montana	1811	0.3	61.1	2446	0.0	69.6	2663	0.3	65.5
Nebraska	2318	0.7	79.8	3227	1.0	67.0	2698	0.7	83.2
Nevada	1845	0.8	67.4	3258	1.4	67.1	2369	0.7	62.8
New Hampshire	1840	0.5	68.5	3376	0.5	79.4	2624	0.4	78.8
New Jersey	2194	2.7	79.7	6006	5.5	89.0	2961	2.5	89.0
New Mexico	2160	0.5	76.6	3767	1.2	97.0	2713	0.5	79.6
New York	2297	6.1	78.9	4795	7.2	83.7	3260	7.2	86.0
North Carolina	2279	3.7	76.9	2519	3.3	54.3	2555	3.7	70.8
North Dakota	2823	0.3	82.6	4601	0.1	82.5	2763	0.3	76.1
Ohio	1599	4.0	64.1	NA	NA	NA	2239	4.0	66.2
Oklahoma	2560	1.3	81.0	2281	0.1	63.9	2643	1.2	82.3
Oregon	1433	0.9	54.1	2653	1.8	67.1	2139	1.0	70.5
Pennsylvania	2522	4.4	80.9	3600	7.9	46.6	2698	4.9	84.9
Rhode Island	2695	0.4	84.3	5001	1.0	98.9	3021	0.4	94.3
South Carolina	2109	1.9	77.1	2561	2.1	36.1	2507	1.9	74.6
South Dakota	2967	0.3	80.9	2191	0.1	60.9	3129	0.3	79.0
Tennessee	2028	2.8	72.3	1650	0.4	54.8	2475	2.8	72.7
Texas	1936	9.6	72.2	2706	8.2	51.0	2566	7.8	81.2
Utah	1512	1.1	49.3	3529	0.6	23.0	2420	0.6	72.6
Vermont	2060	0.2	77.1	2788	0.0	77.3	3098	0.2	87.8
Virginia	2002	2.8	65.7	2707	4.5	67.8	2204	2.1	63.8
Washington	1927	1.9	65.8	4647	2.9	86.8	2699	1.5	81.4
Washington, DC	3279	0.3	83.5	2900	0.6	28.7	3047	0.2	87.6
West Virginia	2461	0.7	74.9	2179	0.3	28.7	2648	0.8	60.9
Wisconsin	1425	1.6	57.4	2107	0.2	67.4	2042	1.7	68.4
Wyoming	1968	0.2	68.7	2068	0.0	65.3	2736	0.1	80.6
Highly concentrated^d^	2194	16.1	82.0	3797	93.7	70.0	2868	74.8	80.0

Abbreviations: HHI, Herfindahl-Hirschman Index; NA, not applicable.

^a^ Data are from IQVIA’s National Prescription Audit PayerTrak for prescriptions dispensed at retail pharmacies in the US. These data do not capture prescriptions filled through mail-order pharmacies. Retail prescriptions (10% out of 3.6 billion claims) paid for through cash, Medicaid fee-for-service, and the Medicaid Children’s Health Insurance Program were excluded because PBM functions, if any, are limited to administrative functions like claims adjudication.

^b^ Concentration is measured by HHI (sum of each PBM’s share of retail prescriptions squared). Certain PBMs (eg, Aetna with Caremark) were combined based on outsourcing and ownership relationships (eMethods in Supplement 1).

^c^ HHI calculation excluded Ohio and Kentucky where, as of 2023, state Medicaid authorities contracted with a single pass-through PBM for all Medicaid managed care business throughout the state.

^d^ Highly concentrated was defined as HHI above 2500. Thirteen states were highly concentrated in commercial insurance and accounted for 16.1% of national commercial retail prescriptions; 37 states were highly concentrated in Medicaid managed care and accounted for 93.7% of national retail prescriptions, with Ohio and Kentucky excluded; and 40 states were highly concentrated in Medicare Part D and accounted for 74.8% of national retail prescriptions.

## Discussion

In this cross-sectional study, PBM market concentration for retail prescriptions varied substantially across states and payer types, with most states having highly concentrated markets for Part D and/or Medicaid managed care. The majority of retail prescriptions for Part D and Medicaid managed care were filled in states with highly concentrated PBM markets where the top 3 PBMs dominate, suggesting PBM reform in publicly funded payer markets should be a priority. These findings can guide federal and state policy to improve PBM accountability and transparency. For example, states with highly concentrated PBM markets within Medicaid managed care, including Hawaii, where a state lawsuit was recently filed,^6^ may consider implementing fee-for-service carve outs of the pharmacy benefit, similar to New York. These findings can also inform federal policy interventions, including proposed legislation targeting anticompetitive PBM business practices in Medicare Part D, and the current investigation into PBM conduct.^1^

One study limitation is the lack of information on plan-level contractual arrangements, including specific PBM functions and authority, although the top 3 PBMs exert considerable control over pharmacy networks, cost sharing, and pharmacy reimbursement rates. Additionally, HHI alone does not provide conclusive evidence of anticompetitive PBM business practices. Future research should examine how PBM market concentration influences pharmacy networks, closures, and patient out-of-pocket costs.

## Notes

Supplement 1. eMethods

Supplement 2. Data Sharing Statement
