# Supplementary material for: Pharmacy Benefit Manager Market Concentration for Prescriptions Filled at Retail Pharmacies by State and Payer Type
Source: JAMA Health Forum. 2026 Feb 6;7(2):e256546. doi: 10.1001/jamahealthforum.2025.6546 (PMC12881978; doi:10.1001/jamahealthforum.2025.6546)
Supplement: Supplement 1. — eMethods [file jamahealthforum-e256546-s001.pdf]

Supplemental Online Content

Qato DM, Chen Y, Van Nuys K. Pharmacy benefit manager market concentration for prescriptions filled at retail pharmacies by state and payer type. *JAMA Health Forum*. 2026;7(2):e256546. doi:10.1001/jamahealthforum.2025.6546

eMethods

This supplemental material has been provided by the authors to give readers additional information about their work.

© 2026 Qato DM et al. *JAMA Health Forum*.

eMethods

A. Data source

We used IQVIA's National Prescription Audit PayerTrak, which includes 92% of prescription fills at US retail pharmacies, including information on the processing PBM, payer type (commercial insurance, Medicare Part-D and Medicaid managed care) and the state where the prescription was dispensed. We excluded mail-order prescriptions and retail prescriptions paid for through cash, Medicaid fee-for-service (FFS) because if a PBM is contracted it often involves only administrative functions. We excluded Ohio and Kentucky from Medicaid managed care analyses because those states' Medicaid authorities contracted with a single pass-through PBM for all managed care plans in 2019 and 2021, respectively. Therefore, these analyses capture 90% of total retail prescriptions because approximately 10% are filled through other payment methods for which PBMs are minimally involved.

B. Plan definition

The IQVIA data document insurance information (method of payment and model types) and the name of the processing PBM for each claim. We combined some processing PBMs based on key outsourcing and ownership arrangements: 1) all claims processed by Prime Therapeutics were assigned to Express Scripts because Prime Therapeutics outsourced its drug benefit rebate negotiation and pharmacy network management services to Express Scripts beginning in 2020; 2) all claims processed by Aetna were assigned to Caremark since the insurer Aetna owns the PBM Caremark; 3) all claims processed by Magellan Rx in 2023 were assigned to Express Scripts because Prime Therapeutics acquired Magellan at the end of 2022. It is possible that additional processing PBMs listed are owned or otherwise controlled by larger PBMs on the list, but because we lack information about these relationships, we have treated them as independent entities in the HHI calculation. This would bias our calculated HHI downward; as such, our calculated HHIs represent conservative (lower-bound) estimates of the true HHI.

C. Herfindahl-Hirschman Index (HHI)

For the overall US retail prescription drug market, we calculate the HHI as the sum of each PBM's share of all retail prescriptions processed, squared. We also calculate HHIs for each payer type, and for each state. Thus, the HHI for a plan *p* in a state *s* is

$$HHI_{s,p} = \sum_{i=1}^{I_{s,p}} S_{i,s,p}^2$$

Where $S_{i,s,p}$ represents PBM *i*'s share of retail prescriptions for plan *p* in state *s*, and $I_{s,p}$ is the total number of PBMs for plan *p* in state *s*. The HHI for a state *s* is

$$HHI_s = \sum_{i=1}^{I_s} S_{i,s}^2$$

Where $S_{i,s}$ represents PBM *i*'s share of retail prescriptions in state *s,* encompassing commercial insurance, Medicare Part D, and Medicaid managed care., and I_s is the total number of PBMs operating in these markets in state *s*.

© 2026 Qato DM et al. *JAMA Health Forum*.
