# Supplementary material for: Pharmacy Benefit Manager Market Concentration for Prescriptions Filled at Retail Pharmacies by State and Payer Type
Source: JAMA Health Forum. 2026 Feb 6;7(2):e256546. doi: 10.1001/jamahealthforum.2025.6546 (PMC12881978; doi:10.1001/jamahealthforum.2025.6546)
Supplement: Supplement 2. — Data Sharing Statement [file jamahealthforum-e256546-s002.pdf]

Data Sharing Statement

Qato. Pharmacy Benefit Manager Market Concentration for Prescriptions Filled at Retail Pharmacies by State and Payer Type. *JAMA Health Forum*. Published February 06, 2026. doi:10.1001/jamahealthforum.2025.6546

Data
Data available: No

Additional Information
Explanation for why data not available: It is proprietary but data can be licensed through IQVIA
